# Prevalence and multidrug-resistant profile of methicillin-resistant *Staphylococcus aureus* and methicillin-resistant *Staphylococcus pseudintermedius* in dogs, cats, and pet owners in Malaysia

**DOI:** 10.14202/vetworld.2023.536-545

**Published:** 2023-03-22

**Authors:** Mohammad Farzad Afshar, Zunita Zakaria, Chen Hui Cheng, Nur Indah Ahmad

**Affiliations:** 1 Department of Paraclinic, Faculty of Veterinary Sciences, Kabul University, 1001, Kabul, Afghanistan; 2 Department of Veterinary Pathology and Microbiology, Faculty of Veterinary Medicine, Universiti Putra Malaysia, 43400, Selangor, Malaysia; 3 Institute of Bioscience, Universiti Putra Malaysia, 43400, Selangor, Malaysia; 4 Department of Companion Animal Medicine and Surgery, Faculty of Veterinary Medicine, Universiti Putra Malaysia, 43400, Selangor, Malaysia

**Keywords:** antimicrobial resistance, antimicrobial susceptibility testing, methicillin resistance, multilocus sequence typing, public health, zoonotic infections

## Abstract

**Background and Aim:**

*Staphylococcus aureus* and *Staphylococcus pseudintermedius* are widespread skin and mucous membrane colonizers and may cause opportunistic infections in humans and animals. This study aimed to identify and characterize methicillin-resistant *S. aureus* (MRSA) and methicillin-resistant *S. pseudintermedius* (MRSP) isolates from domestic and stray dogs and cats and pet owners in Malaysia using molecular epidemiology and antimicrobial profiling.

**Materials and Methods:**

Three hundred and fifty oral and nasal swabs were taken from pet and stray dogs and cats and pet owners; all samples were subjected to culture and biochemical tests and polymerase chain reaction; the selected isolates were put through disk diffusion test and multilocus sequence typing.

**Results:**

One *S. aureus* isolate and three *S. pseudintermedius* isolates were identified as MRSA and MRSP, respectively, of which the MRSA isolate and one of the MRSP isolates showed multidrug resistance and the remaining two MRSP isolates were resistant to one or two antimicrobials. Multilocus sequence typing showed that the MRSA isolate belongs to clonal complex (CC) 789, while for the MRSP isolates, two were in CC45 and one was a singleton.

**Conclusion:**

This study is the first study in Malaysia to perform molecular characterization of MRSP isolated from pet dogs and cats and pet owners. The outcomes of this study reveal that even healthy pet dogs and cats and their owners can be carriers of drug-resistant staphylococci, highlighting the role of pets and pet owners as carriers of MRSA and MRSP in Malaysia.

## Introduction

Human and animal mucous membranes and skin are frequently colonized by *Staphylococcus aureus* and *Staphylococcus pseudintermedius*, which may cause opportunistic infections in the carrier. *Staphylococcus aureus* is the predominant coagulase-positive staphylococci found in humans, with about a quarter of healthy people having long-term colonization of *S. aureus*. On the other hand, *S. pseudintermedius* is the most common coagulase-positive staphylococci detected in healthy cats and dogs. Nevertheless, *S. aureus* can be detected in dogs and cats as well (approximately 20%), especially those living with their human counterparts [1]. In addition, the presence of *S. pseudintermedius* in people who come into contact with animals carrying *S. pseudintermedius* should not be overlooked, especially given the possibility of misidentifying *S. pseudintermedius* as *S. aureus* or *S. intermedius* [1, 2].

As the prevalence of methicillin-resistant *S. aureus* (MRSA) and methicillin-resistant *S. pseudintermedius* (MRSP) increases in the populace, it becomes unavoidable for household animals to be exposed to the bacteria. Methicillin-resistant *S. aureus* is found in a small percentage of healthy canines (0%–4%). Clonal relatedness and typing data showed that MRSA in domesticated animals has been evolving along with MRSA in humans [1]. Detection of MRSA and MRSP in canines and felines suggested that these pathogenic bacteria grow in these animals. Since the incidence of MRSP in humans appears to be low, this indicates the possibility of its zoonotic origin [1, 2]. Both MRSA and MRSP infections in animals can have a detrimental health effect on the animals and humans. Even though most animals carrying MRSA and/or MRSP are asymptomatic, the possibility of opportunistic infections remains. The most common infections include infections of the skin, surgical site, nasal cavity, middle ear, urinary tract, and wound from dog bites. Nonetheless, opportunistic infections at other body sites may also occur [3, 4].

Both *S. aureus* and *S. pseudintermedius* may become resistant to a number of different antimicrobials [5]. In the previous studies conducted in Malaysia, drug-resistant *S. aureus* and *S. pseudintermedius* were isolated from dogs and cats, which were found to be resistant to tetracycline (TE), erythromycin (E), penicillin (P), gentamicin (GEN), and doxycycline (DOXY); in addition, multidrug-resistant MRSA isolates that were intermediately resistant to vancomycin were isolated from humans [6, 7]. Considering *S. aureus* and *S. pseudintermedius* will continue to evolve to become stronger and more resistant to various antimicrobials, monitoring the evolution of *S. aureus* and *S. pseudintermedius* epidemiology has hence become crucial. Regarding this, molecular typing techniques are essential tools for identifying and tracking the clones and lineages of the predominant MRSA and MRSP strains [5–7].

In Malaysia, few studies were conducted to explore the roles of pet dogs and cats as reservoirs of MRSA and MRSP [8–11]. However, no Malaysian studies focused on detecting MRSP from healthy dogs, cats, or pet owners, as well as performing molecular characterization of MRSP isolates. To understand the risks of *S. aureus* and *S. pseudintermedius* transmission between humans and animals, it is necessary to estimate their prevalence and molecular epidemiology [1].

Therefore, this study aimed to estimate the prevalence of *S. aureus* and *S. pseudintermedius* in pet and stray dogs and cats and pet owners, as well as investigate the antimicrobial profile and molecular relationship of the isolates.

## Materials and Methods

### Ethical approval

This study was conducted in accordance with the requirements of the Ethic Committee for Research Involving Human Subjects (approved on August 07, 2020). The animal study protocol was approved by the Institutional Ethics Committee of the Institutional Animal Care and Use Committee (AUP 101; approved on July 03, 2020).

### Study period and location

This study was conducted as a cross-sectional study from August 2020 to March 2021. Stray dogs and cats were selected using convenient sampling from central region of Peninsular Malaysia. Meanwhile, pet dogs and cats attended elective procedures at a Veterinary Hospital.

### Data source and inclusion criteria

In this study, 150 oral swab samples were collected from pet and stray dogs, 100 oral swab samples from pet and stray cats and 100 nasal swab samples were taken from pet owners. Animals that had not received antimicrobial agents for at least 14 days before sampling and had no visible signs of pyoderma, sneezing, and nasal discharge were included in this study, with the exception of stray cats and dogs as there was no history of antimicrobial intake. In contrast to the previous studies in Malaysia that focused on ill pets, this study included only apparently healthy animals to investigate the presence of MRSA and MRSP in the animals, if any, and to explore the role of healthy animals as reservoirs of resistant staphylococci in Malaysia [6, 7].

### Isolation and identification of methicillin-resistant *S. aureus* and methicillin-resistant *S. pseudintermedius*

The collected nasal and oral swabs were placed in Brain Heart Infusion broth (Oxoid™, Basingstoke, United Kingdom) supplemented with 6.5% sodium chloride for 24 h incubation at 35°C. After the incubation, a loopful of the suspension was transferred onto oxacillin (OXN) resistance screening agar base (ORSAB, Oxoid™) and incubated at 35°C for 24 h; the blue and white-pale blue colonies formed were presumptively identified as MRSA and MRSP, respectively [12]. Coagulase test, catalase test, DNase test, and Gram staining were used to differentiate coagulase-positive staphylococci from others [6, 7].

### Deoxyribonucleic acid (DNA) extraction and amplification of targeted genes

Fresh overnight cultures of isolates were used to obtain genomic DNA with the boiling method [13]. The presence of the *nuc*A gene was used to confirm the presumptive *S. aureus* and *S. pseudintermedius* isolates. A thermal cycler (Eppendorf^®^ pro S, Hamburg, Germany) was used for *nuc*A gene amplification, with polymerase chain reaction (PCR) cycling conditions as described earlier by Baron *et al*. [14] and Sasaki *et al*. [15]. To confirm methicillin resistance in the isolates, the presence of the *mec*A gene was examined through PCR amplification, with PCR thermal cycling conditions as described in the study of Strommenger *et al*. [16]. The list of oligonucleotides used in this study is shown in Table-1 [14–16]. *Staphylococcus aureus* ATCC 25923 and *S. pseudintermedius* CCUG 49543 were used as positive controls in the PCR.

**Table-1:** Oligonucleotide sequence for confirmation of *S. aureus*, *S. pseudintermedius*, and methicillin resistance.

Primer	Oligonucleotide sequence (5′-3′)	Size (bp)	References
SA*nuc*A F	TGC TAT GAT TGT GGT AGC CAT C	420	[14]
SA*nuc*A R	TCT CTA GCA AGT CCC TTT TCC A		
Pse *nuc*A F	TRG GCA GTA GGA TTC GTT AA	926	[15]
Pse *nuc*A R	CTT TTG TGC TYC MTT TTG G		
*mec*A F	AAA ATC GAT GGT AAA GGT TGG C	532	[16]
*mec*A R	AGT TCT GCA GTA CCG GAT TTG C		

*S. aureus=Staphylococcus aureus,* *S. pseudintermedius=Staphylococcus pseudintermedius*

The disk diffusion method, as stated in the clinical laboratory and standards, was used to test the antimicrobial susceptibility of the isolates [17]. The isolates were tested against ten antimicrobial agents, namely, OXA (1 µg), enrofloxacin (ENR, 5 µg), GEN (10 μg), rifampicin (RIF, 5 μg), E (15 μg), DOXY (30 μg), trimethoprim-sulfamethoxazole (SXT, 25 μg), chloramphenicol (C, 30 µg), cefoxitin (FOX, 30 μg), and clindamycin (CLN, 2 mcg) (Oxoid™). *Staphylococcus aureus* ATCC 25923 was used as the positive control in antimicrobial susceptibility testing.

### Multilocus sequence typing (MLST)

Multilocus sequence typing of MRSA and MRSP was performed using seven housekeeping genes, as described by Enright *et al*. [18] and Solyman *et al*. [19]. The genomic DNA of the isolates was extracted using the DNeasy Blood and Tissue DNA purification kit (Qiagen^®^, Germantown, Maryland, USA). The PCR products were subjected to Sanger sequencing, and the nucleotide sequences of the seven housekeeping genes were analyzed using Molecular Evolutionary Genetics Analysis version 7.0 software (Pennsylvania State University, USA) and the MLST database (https://pubmlst.org/). In addition, the Multiple Sequence Comparison by Log-Expectation (https://www.ebi.ac.uk/Tools/msa/muscle/) was used to match the sequences. The sequences were also compared with known housekeeping genes in the database for the assignment of allele numbers and sequence types (STs).

The clonal complexes (CCs) of the isolates and the minimum spanning tree were constructed using the goeBURST algorithm (Instituto de Engenharia de Sistemas e Computadores, Lisbon, Portugal) in the PHYLOViz (Instituto de Microbiologia and Instituto de Medicina Molecular, Lisbon, Portugal) [20]. The MRSA isolates that shared six of the seven alleles were assigned to the same CC. The MRSP ST that linked with more than three single locus variants (SLVs) was identified as the main group founder, and both SLVs and double locus variants were included in a CC, as previously described by Damborg *et al*. [21]. Descriptive statistics were used for data analysis.

## Results

### Identification of bacterial isolates

Of the 350 samples collected from dogs, cats, and pet owners, only 17 (4.85%) were positive for *S. pseudintermedius* and 7 (2.00%) for *S. aureus*. Among the seven samples that were positive for *S. aureus*, two were from pet cats (4% of pet cat samples), two were from pet owners (2% of pet owner samples), and three were from stray cats (6% of stray cat samples). No *S. aureus* was detected in pet and stray dogs. Only one of the *S. aureus* isolates from pet cats (2% of the pet cat samples) was identified as MRSA (76_C_M), and the rest were negative for methicillin resistance. Among all the samples, six (6.0%) pet owners, seven (9.3%) pet dogs, and four (8.0%) pet cats were colonized with *S. pseudintermedius*. No positive *S. pseudintermedius* isolates were detected in either stray dogs or cats. Three *S. pseudintermedius* isolates were confirmed as MRSP, of which one (1.3%) was isolated from pet dogs (88_D_F), one (2.0%) was from pet cats (65_C_F), and one (1.0%) was from pet owners (18_W_M).

### Antimicrobial susceptibility testing

The results of the drug susceptibility testing showed that 8 of the 20 methicillin-susceptible *S. aureus* and *S. pseudintermedius* isolates (40%) did not exhibit multidrug resistance, of which 1 was isolated from dogs (1/20, 5%), 4 was from pet owners (4/20, 20%), and 3 was from cats (3/20, 15%). In other words, the remaining 12 isolates (12/20, 60%) were multidrug-resistant, where 5 of the isolates were from dogs (5/20, 25%), 4 were from cats (4/20, 20%), and 3 were from pet owners (3/20, 15%).

**Figure-1:**
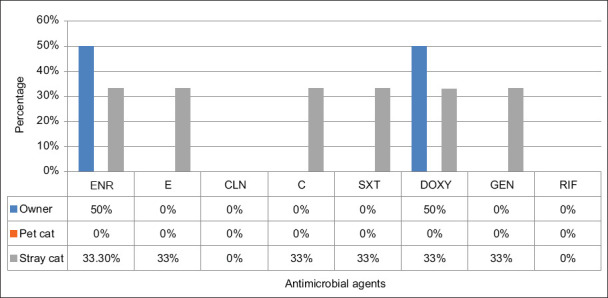
Antimicrobial resistance profile of methicillin-susceptible *Staphylococcus aureus* isolates. ENR: Enrofloxacin (5 µg); E: Erythromycin (15 µg); CLN: Clindamycin (2 µg); C: Chloramphenicol (30 µg); SXT: Trimethoprim-sulphamethoxazole (25 µg); DOXY: Doxycycline (30 µg) GEN: Gentamicin (10 µg); RIF: Rifampicin (5 µg).

**Figure-2:**
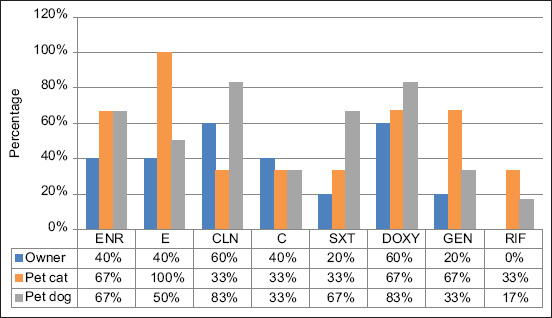
Antimicrobial resistance profile of methicillin-susceptible *Staphylococcus pseudintermedius* isolates. ENR: Enrofloxacin (5 µg); E: Erythromycin (15 µg); CLN: Clindamycin (2 µg); C: Chloramphenicol (30 µg); SXT: Trimethoprim-sulphamethoxazole (25 µg); DOXY: Doxycycline (30 µg) GEN: Gentamicin (10 µg); RIF: Rifampicin (5 µg).

Most isolates (62.5%) from pet owners were resistant to DOXY, whereas none of the isolates from pet owners had resistance to RIF. The *S. aureus* isolates from stray cats showed 100% susceptibility to CLN and RIF but were 33.3% resistant to other antimicrobials. Methicillin-susceptible *S. aureus* isolates from owners showed (1/2, 50%) resistance against DOXY and ENR (Figure-1). Most *S. pseudintermedius* isolates (5/6, 83%) from pet dogs were resistant to DOXY and CLN, whereas the lowest resistance was against RIF (1/6, 17%). All the *S. pseudintermedius* isolates from pet cats were resistant to E 3/3, (Figure-2). For both *S. aureus* and *S. pseudintermedius* isolates, no co-occurrence was observed among pet owners and pets.

For the four methicillin-resistant isolates, the MRSA isolate from the pet cat was resistant to three antimicrobials, namely ENR, GEN, and FOX. On the other hand, two MRSP isolates were resistant only to DOXY, while one MRSP isolate was resistant to multiple antimicrobials.

### Multilocus sequence typing

**Figure-3:**
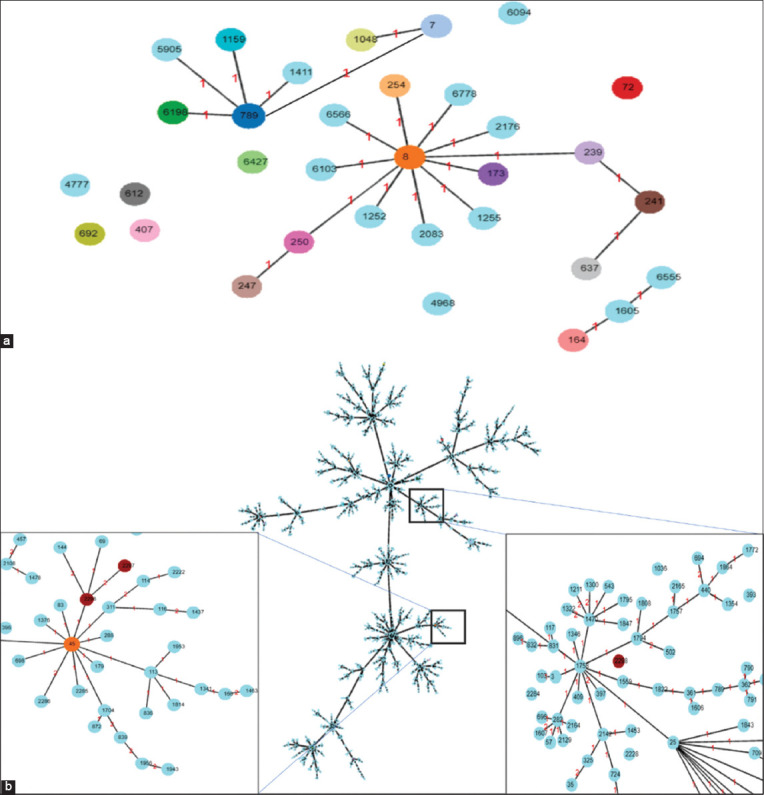
Population snapshot of related sequence types of (a) methicillin-resistant *Staphylococcus aureus* and (b) methicillin-resistant *Staphylococcus pseudintermedius*.

In this study, the only MRSA isolate, 76CM, was identified as ST789 and assigned to CC789 (Figure-3a). For the MRSP isolates, isolate 65CF was identified as ST2296 and belonged to CC45, isolate 18WM was identified as ST2297, a related ST of ST2296, and isolate 88DF was identified as ST2298, a singleton isolated from the dog sample (Figure-3b). It is worth noting that ST789 (MRSA) is the first ST reported in Malaysia, and ST2296, ST2297, and ST2298 (MRSP) are novel STs.

## Discussion

The findings of this study indicate that not only can *S. aureus* colonize and transmit between stray and pet cats and humans, but they can also be methicillin-resistant strains. These findings are in line with the previous studies conducted in Malaysia and other geographic regions of the world. For instance, the detection of MRSA in dogs, cats, and veterinary personnel was reported in Poland, Bangladesh, Egypt, and Iran [22, 23, 24, 25]. However, the prevalence of MRSA (1/50, 2.00% of pet cat samples) in this study was lower than the prevalence reported by Aklilu *et al*. [6] (5/50, 10% of dog samples; 3/50, 6.00% of cat samples), Ghani *et al*. [26] (7/55, 12.73% of stray cat samples), and Paul *et al*. [27] (10/129, 7.80% of dog samples).

The difference in the prevalence of MRSA may be due to several reasons. The first reason could be the difference in sampling sites. The samples in this study were collected only from the oral cavity because, according to reports, *S. aureus* and *S. pseudintermedius* are most commonly found in this area and dual carriage of *S. aureus* and *S. pseudintermedius* was detected in this area as well [28]. On the other hand, the samples in the mentioned studies were taken from the nasal cavity, oral cavity, axilla, and perineum. Furthermore, Beck *et al*. [29] reported that *S. aureus* was found in the nasal cavity and rectum, and *S. pseudintermedius* was detected in the rectum of dogs. Another factor that may affect the prevalence of MRSA or MRSP is the inclusion criteria for sample collection. In the study by Aklilu *et al*. [6], samples were gathered from inpatient and outpatient animals, whereas the samples collected in this study were from apparently healthy dogs and cats only. The previous studies showed that the prevalence of MRSA or MRSP tends to be higher in animals with skin problems. For example, Beck *et al*. [29] isolated 70 (40.5%) MRSP and 3 (1.7%) MRSA isolates from the skin cultures of 172 dogs with pyoderma. The study of Loncaric *et al*. [30] reported that most of the MRSA isolates were isolated from wounds, fistula, dermatitis, abscess, and sinus.

In this study, MRSP was detected in pet dogs, pet cats, and their owners but at an overall low prevalence of 0.9%. Similar findings were reported by previous studies in which 4.3% and 2.8% of pet owners and dogs with pyoderma were MRSP positive [31, 32]. In contrast, a higher prevalence of *S. pseudintermedius* was reported by Mohamed *et al*. [7] and Worthing *et al*. [33], which was 11.5% (23/200) of dogs and cats in Malaysia and 8.0% (4/53) of personnel-owned dogs in Australia, respectively. Moreover, Beck *et al*. [29] reported that MRSP prevalence in dogs was as high as 26.7% in dogs recovered from pyoderma in the dermatological ward in the dermatological ward.

The lower prevalence of MRSP in this study could be due to the selection of sampling sites. As mentioned before, this study only collected samples from nasal and oral cavities. However, more sampling sites were included in the mentioned studies, including oral and nasal cavities, skin, and rectum. Another possible cause for the lower prevalence of MRSP in this study could be the health condition of the cats, dogs, and pet owners. A previous study showed that there is a high chance of detecting MRSP in sick humans and diseased animals compared to those that are healthy [34]. For example, Mouney *et al*. [35] reported a low prevalence of methicillin-resistant staphylococci at 1.6% in healthy dogs.

It seems that transmission of *S. pseudintermedius* from pets to humans is also possible. A study in Taiwan detected *S. pseudintermedius* in a child with hemophilia who had a history of raising two dogs [36]. This finding demonstrated the possibility of pet-owner transmission of *S. pseudintermedius* as this bacterium is a normal inhabitant of the ear and skin of dogs [37]. In this study, all *S. pseudintermedius* isolates were isolated from healthy pets and owners that did not visit a hospital or had any surgery in the past 6 months before sampling. These pet owners were having close contact with their pet dogs, and hence, were exposed to the possibility of transmission of MRSA or MRSP from their pets.

In this study, most *S. pseudintermedius* isolates from pet dogs and cats and pet owners were resistant to TE, lincomycin, and macrolide classes of antimicrobials, which are common antibiotic classes used as human medicines, while susceptible to RIF. Similarly, the *S. pseudintermedius* isolates from dogs and cats in Australia did not exhibit RIF resistance, while 20.0% and 0.4% of the isolates exhibited DOXY and CLN resistance, respectively [38]. Furthermore, the percentage of *S. pseudintermedius* isolates that exhibited resistance against C in this study (33%) is comparable with the study conducted in Spain (25%), whereas the resistance against SXT (12.5%) and E (31.1%) reported in Spain was lower [1].

Both *S. aureus* isolates from stray cats were susceptible to CLN and RIF. This finding is similar to the finding reported in a study conducted in Spain, in which resistance against CLN was observed in some of the *S. aureu*s isolates [1]. In the veterinary hospital, where this study was done the usage of RIF is infrequent, while DOXY, SXT, and ENR are used in routine treatment of small animal cases (CH Cheng, personal communication). Therefore, the resistance to RIF did not occur.

In this study, the MRSA isolates detected from pet cats was resistant to three antimicrobials, namely ENR, GEN, and FOX (Table-2). This finding is similar to the findings of other studies conducted in Malaysia and neighboring countries (such as Singapore), which observed resistance against OXA, ENR, and board-spectrum antimicrobials among the MRSA isolates [39].

**Table-2:** Antimicrobial resistance profile of *S. aureus* and *S. pseudintermedius* isolates.

Sample ID	Name of isolate	Sample	Antimicrobial resistance profile
77SP	*S. pseudintermedius*	Pet owner	ENR, E, CLN, C, SXT, DOXY, GEN
90SP	*S. pseudintermedius*	Pet dog	ENR, E, CLN, SXT, DOXY, GEN
63SP	*S. pseudintermedius*	Pet dog	ENR, CLN, SXT, DOXY, GEN
98SP	*S. pseudintermedius*	Pet dog	ENR, E, CLN, C, DOXY, RIF
68SP	*S. pseudintermedius*	Pet owner	CLN, DOXY
66SP	*S. pseudintermedius*	Pet cat	ENR, E, CLN, DOXY, GEN
44SP	*S. pseudintermedius*	Pet owner	ENR, E, CLN, C, DOXY
74SP	*S. pseudintermedius*	Pet dog	E, CLN, C, SXT, DOXY
23SP	*S. pseudintermedius*	Pet cat	E, C, SXT, DOXY, RIF
1SA	*S. aureus*	Pet cat	Susceptible to all
4SA	*S. aureus*	Stray cat	C
7SA	*S. aureus*	Stray cat	Susceptible to all
10 SA	*S. aureus*	Stray cat	ENR, E, SXT, DOXY, GEN
125SA	*S. aureus*	Pet owner	ENR, DOXY
126SA	*S. aureus*	Pet owner	Susceptible to all
101SP	*S. pseudintermedius*	Pet dog	DOXY, OXA
36SP	*S. pseudintermedius*	Pet owner	CLN, OXA
56SP	*S. pseudintermedius*	Pet owner	CLN, OXA
26 SP	*S. pseudintermedius*	Pet cat	ENR, E, GEN
150SP	*S. pseudintermedius*	Pet Dog	ENR, CLN, SXT, DOXY
18WM	*S. pseudintermedius*	Pet owner	DOXY
76CM	*S. aureus*	Pet cat	ENR, GEN, FOX
88DF	*S. pseudintermedius*	Pet dog	DOXY, OXA
65CF	*S. pseudintermedius*	Pet cat	ENR, DOXY, GEN, OXA

ENR=Enrofloxacin (5 μg), E=Erythromycin (15 μg ),CLN=Clindamycin (2 μg); C=Chloramphenicol (30 μg),SXT=Trimethoprim-sulfamethoxazole (25 μg), DOXY=Doxycycline (30 μg), GEN=Gentamicin (10 μg), RIF=Rifampicin (5 μg), FOX=Cefoxitin (30 μg), OXA=Oxacillin (1 μg), *S. aureus=Staphylococcus aureus, S. pseudintermedius=Staphylococcus pseudintermedius*

The MRSP isolates detected from pet dogs and cats were resistant to TE, fluoroquinolone, aminoglycoside, and P classes of antimicrobials. This finding is in line with the study by Gagetti *et al*. [40], which reported that all the isolated *S. pseudintermedius* were resistant to OXA and TE. Similarly, a study in Singapore also indicated that the *S. pseudintermedius* isolates detected from pet dogs and cats were methicillin-resistant, and 78% (40/51) of the isolates turned out to be multidrug-resistant [39]. However, it is worth noting that the *S. pseudintermedius* isolates in another study conducted in Malaysia did not show resistance against OXA [7], which may imply that methicillin resistance is playing a role in the development of multidrug resistance in *S. pseudintermedius*

In this study, an MRSP isolate from pet owners (18_W_M), which was positive for the *mec*A gene, did not show resistance against OXA. A similar finding was reported in the study conducted by Fabri *et al*. [41]; that is, OXA-susceptible MRSA was isolated from nasal cavities of healthy dogs and their caretakers. While the reasons behind the susceptibility to OXA in MRSA remain unclear, some possible explanations include: (1) The presence of the transposable element IS1181 in the *mec*A gene, which leads to the suppression of PBP2a expression; (2) deletion of a single nucleotide from the area that codes for the N-terminal of PBP2a; (3) suppression of the *mec*A gene through the plasmid-encoded non-functional N-terminally truncated blaR1 sensor-transducer gene, even in the existence of beta-lactams; and (4) cell wall production is affected by mutations in the *fem*XAB genes, which encode proteins tangled in pentaglycine bridge production.

Incorrect or overutilization of antimicrobials in different geographical regions plays a significant part in the development of resistance in bacteria. The drug resistance in the isolates in this study may be due to the use of these drugs on the recruited pets or their owners [22]. Therefore, to slow down the development progress of multidrug resistance among bacteria and reduce the chance of spreading drug-resistant bacteria in the community, both doctors and pet owners must practice prudent use of antimicrobials and abide by self-hygiene, including performing an antibiogram before prescribing antimicrobials. In addition, resistance may also develop from mutations during bacterial clonal expansion and the transfer of antimicrobial resistance genes among bacteria from the same or different species [42]. The low resistance against RIF among *S. pseudintermedius* isolates could be due to the ability of RIF to penetrate the cell wall, making it an effective antimicrobial even to MRSP [43].

Antimicrobial resistance was announced as a global challenge by the World Health Organization in 2001, and it has been reported that antimicrobial resistance costs more than one billion euros per year [44]. Therefore, the one health approach was proposed to cope with the destructive effects of antimicrobial resistance on humans, animals, and environments. Conducting public campaigns regarding the effects of irrational use of antimicrobials; improving living standards, hygiene measures, and health-care systems; reducing irrational use of antimicrobials to decrease the spread of antimicrobial agents to the environment; conducting local, national, and international antimicrobial resistance surveillance and research regarding the mechanisms behind the antimicrobial resistance; finding and promoting novel and reliable diagnostic techniques and vaccines; training of microbiologists, epidemiologists, and infectious diseases specialists; supporting research for finding new ways of treatment; and finally, including the antimicrobial resistance issue in political agenda are some of the strategies that can be used to battle against antimicrobial resistance [45].

The MLST analysis conducted in this study identified the MRSA isolate (76CM) from healthy pet cats as ST789. This ST is part of CC789, with several related STs as shown in Figure-3. The STs related to ST789 include ST1159 (isolated from the nasal cavity of a human in Switzerland), ST5905 (from unknown sources in the United Kingdom), ST1411 (from the Netherlands), ST6198 (from a human wound in Myanmar), and ST7 (from blood in Thailand, from nasal cavity in the United States, from Switzerland).

To the best knowledge of the authors, this is the first ST789 reported in Malaysia. Nevertheless, this ST was also detected in several other countries, such as Pakistan (from human pus), the Philippines (from humans), Japan (from bovine mastitis, in milk), Ghana, and Kenya (SCC*mec* type IV and Panton-Valentine leukocidin positive) [46, 47, 48]. This suggests that ST789 usually originates from humans or the environment, causing local or systematic MRSA infections. However, in this study, isolate 76CM was isolated from an apparently healthy 1-year-old cat. Since, according to MLST and SCC*mec* typing, ST789 is a community-associated strain (SCC*mec* type V), the cat may have acquired MRSA from its owner or the environment.

The MRSP isolates, 65CF and 18WM, were assigned new STs, ST2296 and ST2297, respectively, by the PubMSLT curated by Vincent Perreten. These STs are novel STs, and they are related to each other. ST2296 belongs to CC45, a worldwide spread CC with members in Europe, the Middle East, Asia, and Finland [49]. There were two STs related to ST2296, that is, ST69 and ST144 (methicillin-susceptible *S. pseudintermedius* isolated from dogs in the United States). Similar to ST69 and ST144, ST2297 was also isolated from a pet owner, but it was resistant to methicillin. Based on the PubMLST database, there were other STs in CC45 detected in other countries: Methicillin-resistant ST1376 isolated from dog skin in the Netherlands; community-acquired, methicillin-susceptible ST698 isolated from a dog with otitis in the Netherlands; methicillin-susceptible ST2286 isolated from dogs with pyoderma and otitis and healthy human in Brazil; and methicillin-resistant ST179 isolated from a cat with pyoderma in Israel. Further query in the PubMLST database [50] indicated that ST311, a related ST of CC45, was isolated from a healthy human carrier in Thailand, which is similar to ST2297 which was isolated from a healthy pet owner. ST2298 (88DF) isolated from a dog in this study was a singleton, a triple locus variant from ST1798, which was isolated from a dog with pyoderma in China.

In Israel and Thailand, the bacteria from the ST45 clonal lineage are the predominant strains [46]. Furthermore, ST45 was detected in dogs and cats with pyoderma in Australia [47]. Based on the resistance genes, isolates typed as ST45 in Thailand are similar to isolates reported from Europe and North America [51]. Besides, non-typeable SCC*mec* STs belonging to CC45 were also isolated from symptomatic dogs and cats in Italy and clinical specimens from dogs in Italy and Netherlands [52, 53]. These findings suggest the possibility of the transmission of ST2296, ST2297, and ST2298 from overseas to Malaysia through the entry of infected animals or humans from Southeast Asian and European countries.

Although MRSA and MRSP can occur in both healthy and diseased animals and humans, the prevalence is higher in diseased animals. In the present study, even though MRSA and MRSP were detected in apparently healthy dogs, cats, and pet owners, the prevalence was low. The absence of MRSP isolates among dogs and cats from the shelter and the low prevalence of MRSA among this group suggest that cats and dogs in contact with humans are more likely to acquire MRSA and MRSP than those without contact with humans. Therefore, screening and identification of MRSA and MRSP in inpatient animals and their owners are required to understand the prevalence of these bacteria among them in the future. The results of antimicrobial susceptibility testing in this study indicated that both methicillin-resistant and methicillin-susceptible isolates could be multidrug-resistant, which is a warning for the community and practitioners.

## Conclusion

Each MRSA and MRSP isolates from animals and humans belong to a different ST, which were also found in other countries in Asia and Europe. Most of the isolated *S. aureus* and *S. pseudintermedius* were multidrug-resistant, which is an alarm for the public regarding the significance of pets as reservoirs of antimicrobial-resistant bacteria. As the first study investigating the molecular typing of MRSP in Malaysia, this study could be a reference for future studies on MRSP. As the number of antimicrobial-resistant bacteria is increasing worldwide, the need for devising and promoting new strategies, treatments, and drugs has become imperative. Hence, the One Health approach, which is a way of utilizing different strategies such as reducing unnecessary use of antimicrobials and improving hygiene measures, is recommended in the practice of medicine to slow down the development and spread of antimicrobial-resistant bacteria.

## Authors’ Contributions

ZZ, CHC, NIA, and MFA: Conceptualization. ZZ, CHC, NIA, and MFA: Methodology. MFA and CHC: Data collection and analysis. ZZ, CHC, and NIA: Supervised the study. MFA: Writing the original draft. ZZ, CHC, NIA, and MFA: Review and editing of the manuscript. All authors have read, reviewed, and approved the final manuscript.
